# Using absolutist word frequency from online searches to measure population mental health dynamics

**DOI:** 10.1038/s41598-022-06392-4

**Published:** 2022-02-16

**Authors:** Jais Adam-Troian, Eric Bonetto, Thomas Arciszewski

**Affiliations:** 1 School of Psychology, Keele University, Keele, Newcastle, ST5 5BG UK; 2 Department of Psychology and Education, Aix-Marseille University, Marseille, France

**Keywords:** Psychology, Diagnostic markers, Predictive markers

## Abstract

The assessment of population mental health relies on survey data from representative samples, which come with considerable costs. Drawing on research which established that absolutist words (e.g. never) are semantic markers for depression, we propose a new measure of population mental health based on the frequency of absolutist words in online search data (absolute thinking index; ATI). Our aims were to first validate the ATI, and to use it to model public mental health dynamics in France and the UK during the current COVID-19 pandemic. To do so, we extracted time series for a validated dictionary of 19 absolutist words, from which the ATI was computed (weekly averages, 2019–2020, *n* = 208) using Google Trends. We then tested the relationship between ATI and longitudinal survey data of population mental health in the UK (n = 36,520) and France (n = 32,000). After assessing the relationship between ATI and survey measures of depression and anxiety in both populations, and dynamic similarities between ATI and survey measures (France), we tested the ATI’s construct validity by showing how it was affected by the pandemic and how it can be predicted by COVID-19-related indicators. A final step consisted in replicating ATI’s construct validity tests in Japan, thereby providing evidence for the ATI’s cross-cultural generalizability. ATI was linked with survey depression scores in the UK, *r* = 0.68, 95%CI[0.34,0.86], *β* = 0.23, 95%CI[0.09,0.37] in France and displayed similar trends. We finally assessed the pandemic’s impact on ATI using Bayesian structural time-series models. These revealed that the pandemic increased ATI by 3.2%, 95%CI[2.1,4.2] in France and 3.7%, 95%CI[2.9,4.4] in the UK. Mixed-effects models showed that ATI was related to COVID-19 new deaths in both countries *β* = 0.14, 95%CI[0.14,0.21]. These patterns were replicated in Japan, with a pandemic impact of 4.9%, 95%CI[3.1,6.7] and an influence of COVID-19 death of *β* = 0.90, 95%CI[0.36,1.44]. Our results demonstrate the validity of the ATI as a measure of population mental health (depression) in France, the UK and to some extent in Japan. We propose that researchers use it as cost-effective public mental health “thermometer” for applied and research purposes.

## Introduction

The ongoing COVID-19 pandemic context has generated global consequences for public mental health^1^. Growing evidence from longitudinal studies suggests worldwide increases in PTSD, depression and anxiety disorders in 2020 compared to previous years^2–5^. Research suggests that these consequences result from the combination of lockdown measures (i.e. social isolation)^6,7^, economic downturn^8^ and fear of the pandemic itself^9,10^. In the US for instance, depression levels increased significantly during the pandemic (April 2020), but even more so among young adults (age 18–34)^11^. Understanding and monitoring the extent to which the pandemic affects public mental health is therefore critical to prevent indirect increases in suicide and substance abuse-related deaths during pandemics^12,13^.

Proper monitoring of public mental health would allow to quantify the ‘side-effects’ of policies aimed to slow down the spread of COVID-19^14^, enabling to factor those in cost–benefit analyses to assist decision making. However, current techniques to monitor population mental health rely heavily on self-report data^2–10^. This is problematic because longitudinal surveys conducted on representative samples of any population are costly to implement, and necessarily involve complex methodological challenges (such as dealing with attrition^15^). More importantly maybe, longitudinal survey studies usually provide a handful of time points and are often conducted in reaction to events, which prevent the use of more powerful analytical techniques for causal inference^16,17^.

To tackle these issues, we propose a novel behavioral measure of public mental health that allows for cheap, continuous probing of population mental health levels. To do so, we draw on cognitive theories of depression, which established that absolutist thinking is associated with anxiety, depression and suicidal ideation^18–21^. Accordingly, studies show that absolutist words (e.g. completely, totally, all) are more prevalent among depressed, suicidal patients and in writings of suicidal individuals^18–21^. For instance, clinical research has found that absolutist “dichotomous” thinking style is a strong predictor of depressive relapse^22^. This is because absolutist thoughts, devoid of nuance, increase unfounded perceptions of threats, unrealistic expectations and ultimately foster more maladaptive coping strategies. Hence, having an extreme, absolute cognitive style or though pattern is a key risk factor for anxiety-depression and cognitive rigidity is an important component of psychological models for those disorders^23,24^.

The frequency of absolutist word use in speech is therefore a reliable linguistic marker for depression and related disorders. Recent investigations have leveraged this marker using search engine data (e.g. Google Trends^25^) to show how an index of absolutist word frequency in online searches (absolute thinking index, ATI) can predict state-level suicide rates in the US^26^.

The ATI approach is partly in line with previous research using online search behavior data to attempt to measure population mental health^27,28^ by looking at frequency of search words related to mental health (e.g. OCD, anxiety, suicide). The novelty of this method is that it does not rely on the *meaning* of searched words, but on their *psychological function*^29^. Indeed, the focus on meaning rests on strong untested assumptions, for instance that searching for mental health symptoms is a reliable marker for mental health diagnosis, and generates sensitivity to sentence wording (e.g. inclusion of question mark or not). This has led researchers to question the validity of measures based on online search data^30,31^. In comparison, the ATI’s only assumption is that the way individuals typewrite is influenced by their mood, an uncontroversial assumption which has empirically been verified recently^32^.

In fact, the ATI approach proposes that frequency of absolutist word “slips” in online searches—regardless of the searched topic itself—should be a reliable proxy for population mental health (and depression in particular). Given that absolutist word use indicates higher levels of depression, the likelihood of absolutist word “slips” in search requests should increase with the level of individual depressive symptoms (e.g. typing “is the airport *totally* shut down?” instead of “is the airport shut down?”). Drawing on the ATI method, the present study has therefore a double aim. First, we propose to validate the ATI for the first time—by leveraging survey data from longitudinal studies of mental health during the COVID-19 pandemic in the UK and in France. Second, we use the ATI to model the population mental health dynamic during the pandemic and to quantify its impact.

## Methods

### Ethical statement

The study was conducted in accordance with the 2016 APA Ethical Principles of Psychologists and Code of Conduct. Ethic approval requirement was waived due to the nature of our data (publicly accessible anonymous archival data on aggregates). The data underlying our findings is openly accessible at https://osf.io/2yh65/?view_only=e750deb7ac0a46358cab870e3fb1d260.

### Online search data

To compute the ATI, we used search volume data from Google Trends. In short, Google Trends provides researchers with the *weekly* frequency at which a specific search term is typed, compared to all different search terms across specified languages and geographical areas^25^. These data are publicly available and can be easily retrieved. When extracting data on a specific word in Google Trends it is possible to retrieve its prevalence among all search entries in a given population. For example, data extracted for the word “completely” counts the relative frequency at which this word was typed by the user population among every request in the specified geographical area spanning a given time period.

### Data extraction

The criteria for country selection was (1) survey data availability (2) over a sufficiently long period of time in countries (3) displaying similar pandemic dynamics and government responses for comparability. We therefore focused our efforts on the UK and France, which met all criteria. For each time period in France and in the UK, we proceeded similarly by collecting search volume time series for word searches regarding the 19 terms from the absolutist language dictionary^18,26^ (e.g. “absolutely,” “all,” “never,” see OSF page for the full list).

The data was extracted one term at a time to avoid extremely rare word combinations that would yield to few data (e.g. ‘all’ and ‘absolutely’ and ‘nothing’) as well as to bypass the platform’s word number limitation (n = 5). Whenever data was too few and the output indicated an estimate “ < 1”, we rounded it down to 0 to allow for quantitative analysis (those occurrences were overall rare though, < 1% of total data). In French, the words for “completely” (“complètement’) and “ever” (“déjà”) are written with accents, so we also extracted them without accents because individuals sometimes omit them to type faster. The extracted data thus consisted in 19 time series (21 for France) from which we computed a single average score variable (ATI) after checking for reliability (see OSF page for the list).

For the validation analysis in the UK, we computed the ATI in England from March 23rd to August 9th of 2020 (α = 0.76) to match longitudinal survey data of anxiety and depression collected from the post-lockdown UCL COVID-19 Social Study^32^ (n = 20). This study comprised a final analytic sample of 36,520 individuals, among which 76% were women, with an over-representation of college-degree holders (70%), an under-representation of non-whites (5%), and a slight underrepresentation of individuals with a diagnosed mental illness (18.3%). To correct for these sampling features, researchers weighted the data to reflect UK population proportions. The survey measured anxiety and depression using two well validated tools, the Generalized Anxiety Disorder scale (GAD-7; 4-point responses)^33^ and the Patient Health Questionnaire (PHQ-9; for depression, 4-point responses)^34^. Both scales indicate moderate anxiety/depression from 10 points and on, which is why this value was chosen as a cutoff score to reflect the proportion of individuals having moderate levels of anxiety/depression at each time point.

For this analysis, we also computed a previously used search data-based measure of common mental health symptoms^28^ to examine how it would perform (averaging the following words: “anxiety,” “depression,” “OCD”, “hopeless,” “angry,” “afraid,” “apathy,” “worthless,” “worried,” “restless,” “irritable,” “tense,” “scattered,” “tired,” “avoiding,” “procrastinate,” “insomnia,” “suicidal,” and “suicide”).

Similarly for the validation analysis in France, we computed the ATI (using regional-level data this time; α = 0.74) to match data from the COVIPREV longitudinal survey (see https://www.santepubliquefrance.fr/etudes-et-enquetes/coviprev-une-enquete-pour-suivre-l-evolution-des-comportements-et-de-la-sante-mentale-pendant-l-epidemie-de-covid-19) from the week of March 23rd to that of October 19th of 2020 (16 weekly waves, 12 regions, n = 192). This study comprised a weekly total of 2000 participants over 18 of age (total n = 32,000) recruited through a quota method based on the latest available (2016) French census to generate representative samples each time. These online surveys measured anxiety and depression using the Hospital Anxiety and Depression Scale^35^. As in the UK study, researchers from COVIPREV in France used a cutoff score of 10, so that weekly percentages reflect the proportion of French individuals displaying moderate levels for both anxiety and depression.

In addition to these two analyses, we computed the ATI for the whole of 2019 and 2020 in both countries to conduct a pandemic impact analysis (α = 0.86; n = 208). Finally, we decided to replicate these analyses using a Japanese version of the ATI. This country was chosen due to its cultural and geographical distance to Western Europe^36^, and because the pandemic there displayed different dynamics^37^. This would allow us to provide further evidence for ATI’s cross-cultural validity.

## Results

### Analysis 1: UK validation

**Figure 1:**
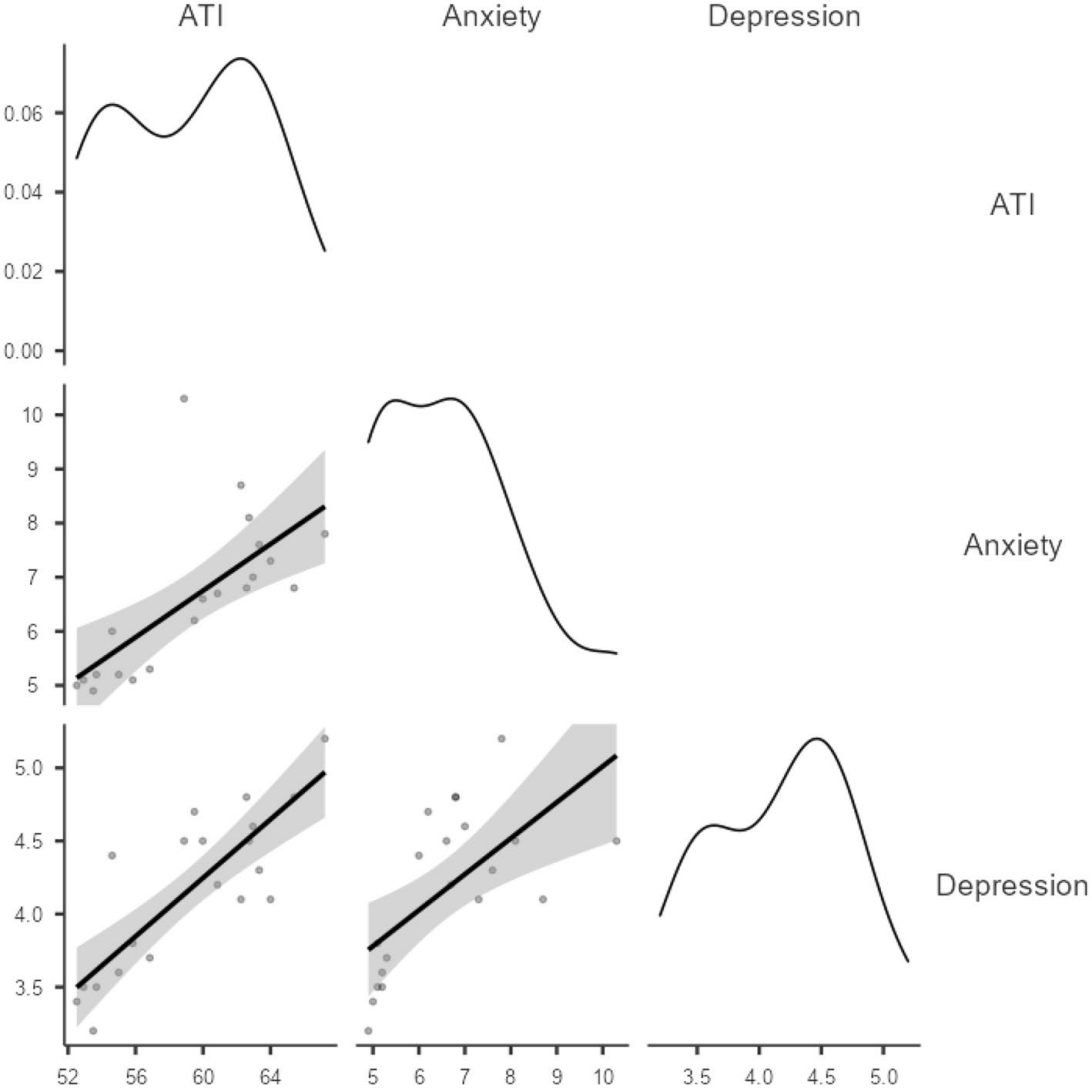
Correlation plots for ATI, survey Anxiety and Depression measures. Grey areas indicate SEs and distribution shapes densities.

Results from Pearson correlation analyses indicated that ATI was linked with survey anxiety, *r*(20) = 0.68, 95%CI[0.34,0.86], *p* < 0.001, as well as depression measures *r*(20) = 0.83, 95%CI[0.60,0.92], *p* < 0.001 (see Fig. 1). ATI was also correlated with average search frequency for mental health-related terms *r*(20) = 0.46, 95%CI[0.03,0.75], *p* = 0.04, although this last measure was not substantially related to either survey anxiety *r*(20) = 0.13, 95%CI[− 0.33,0.54], *p* = 0.59 or depression measures, *r*(20) = 0.34, 95%CI[− 0.13,0.68], *p* = 0.15.

Yet, because our data are essentially time series, we needed to rule out non-independence of observations as a potential source of bias. To do so, we regressed all our measures on time and extracted the remaining residuals of these analyses. All variables exhibited linear decay, *β* =  − 0.82, 95%CI[− 1.10, − 0.54], *p* < 0.001 for ATI, *β* =  − 0.94, 95%CI[− 1.11, − 0.78], *p* < 0.001 for anxiety and *β* =  − 0.71, 95%CI[− 1.06, − 0.36], *p* < 0.001, for depression—at the exception of average frequency of mental health searches *β* = 0.23, 95%CI[− 0.71,0.25], *p* = 0.33. We then re-ran Pearson correlations using the residuals. Although the link between ATI and depression held, *r*(20) = 0.61, *p* = 0.006, the relationship with anxiety reversed, *r*(20) =  − 0.50, *p* = 0.03. Suggesting a potential artefactual origin (i.e. from data non-independence).

This first validation analysis therefore provided evidence for predictive, concurrent and construct validity of the ATI. Not only this measure was strongly linked with survey measure of depression and, but it also displayed a similar dynamic over time than what was found in the UCL COVID-19 Social Study’s results^38^. However, the sample size was small and we still needed to establish cross-cultural validity of the ATI before using it as a proxy for population mental health in France. We therefore performed a second validation.

### Analysis 2: France validation

In France, the larger number of observations and the data structure allowed to proceed with finer grained analyses. The only difference was that depression and anxiety measures in that sample reflect provisional diagnoses for disorders, not scores (i.e. percentage of individual above scale cutoff scores). To account for clustering within regions (n = 12) and to directly incorporate time-related trends (survey waves, n = 16), mixed-effects models were computed for each survey measure allowing both slopes and intercepts to vary according to the following specifications:

1. *depression* = *1* + *ATI* + *wave* + *(1* + *ATI* + *wave | region)*
2. *anxiety* = *1* + *ATI* + *wave* + *(1* + *ATI* + *wave | region)*

Results replicated what we found in the UK, with 1% ATI increase associated with + 0.23% depression diagnoses 95%CI[0.09,0.37], *t*(22) = 3.30, *p* = 0.003, while it was not robustly linked with anxiety *β* = 0.20, 95%CI[− 0.01,0.38], *p* = 0.076 (full model tables are available at https://osf.io/2yh65/?view_only=e750deb7ac0a46358cab870e3fb1d260).

**Figure 2:**
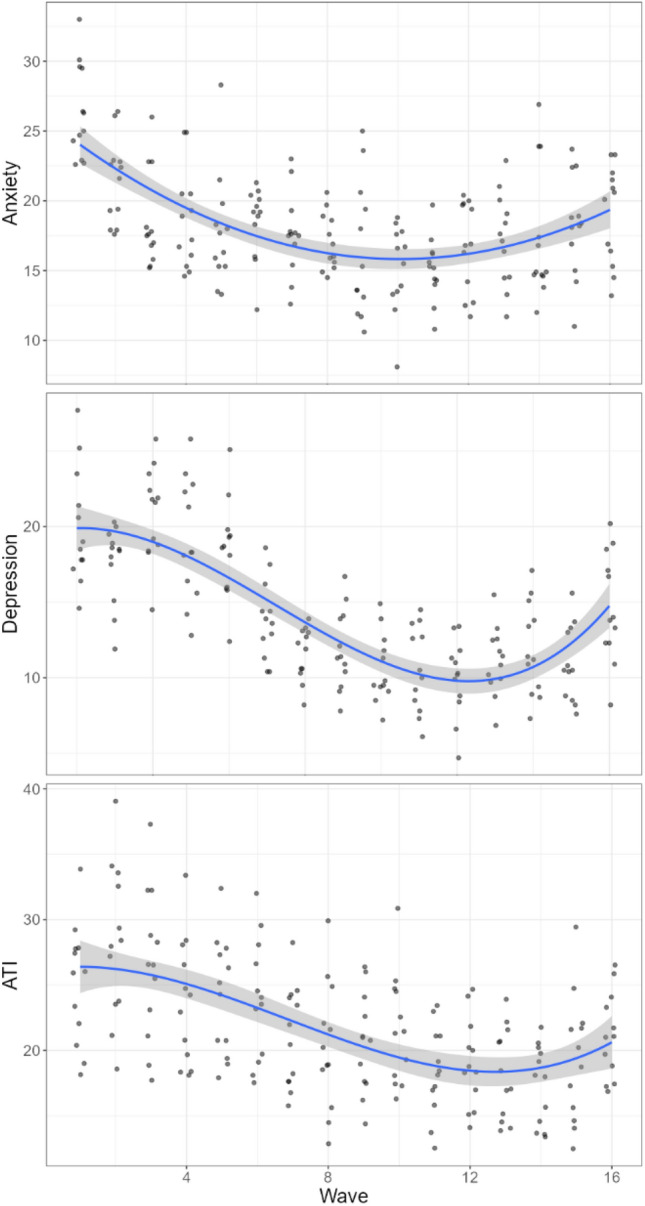
Chronogram for ATI, Anxiety and Depression measures across regions and over the weeks from March 23rd (wave 1) to October 19th (wave 16). Blue fitted lines represent second-order polynomial for Anxiety and third-order for ATI and depression. Grey areas represent SEs.

Interestingly, all three variables seemed to display a polynomial dynamic, with an initial decline and stabilization until week 10 (June 8th) and a progressive increase again (see Fig. 2). This was confirmed by further mixed-modelling according to the following specifications:

(3) *depression* = *1* + *wave* + *wave*^*2*^ + *wave*^*3*^* (1 | region)*
(4) *anxiety* = *1* + *wave* + *wave*^*2*^ + *wave*^*3*^* (1 | region)*
(5) *ATI* = *1* + *wave* + *wave*^*2*^ + *wave*^*3*^* (1 | region)*

Although anxiety rates displayed a second-order (quadratic) trend, *β* = 0.22, 95%CI[0.07,0.35], *p* = 0.005 (cubic parameter *p* > 0.10, see full model on the OSF project page), both ATI and depression rates displayed similar cubic behavior with respectively *β* = 0.01, 95%CI[0.006,0.013], *p* < 0.001 and *β* = 0.02, 95%CI[0.01,0.03], *p* < 0.001. This provided further evidence for the specificity of ATI—which mimicked depression dynamics, and the polynomial trend observed was indicative of something ongoing around the same time the 2nd wave of the pandemic started in France. Having established the relevance of using ATI as a proxy for mental health (and more specifically depression) in both the UK and France, we proceeded to our main analysis of the pandemic’s effect on population mental health.

### Analysis 3: Joint pandemic impact analysis

**Figure 3:**
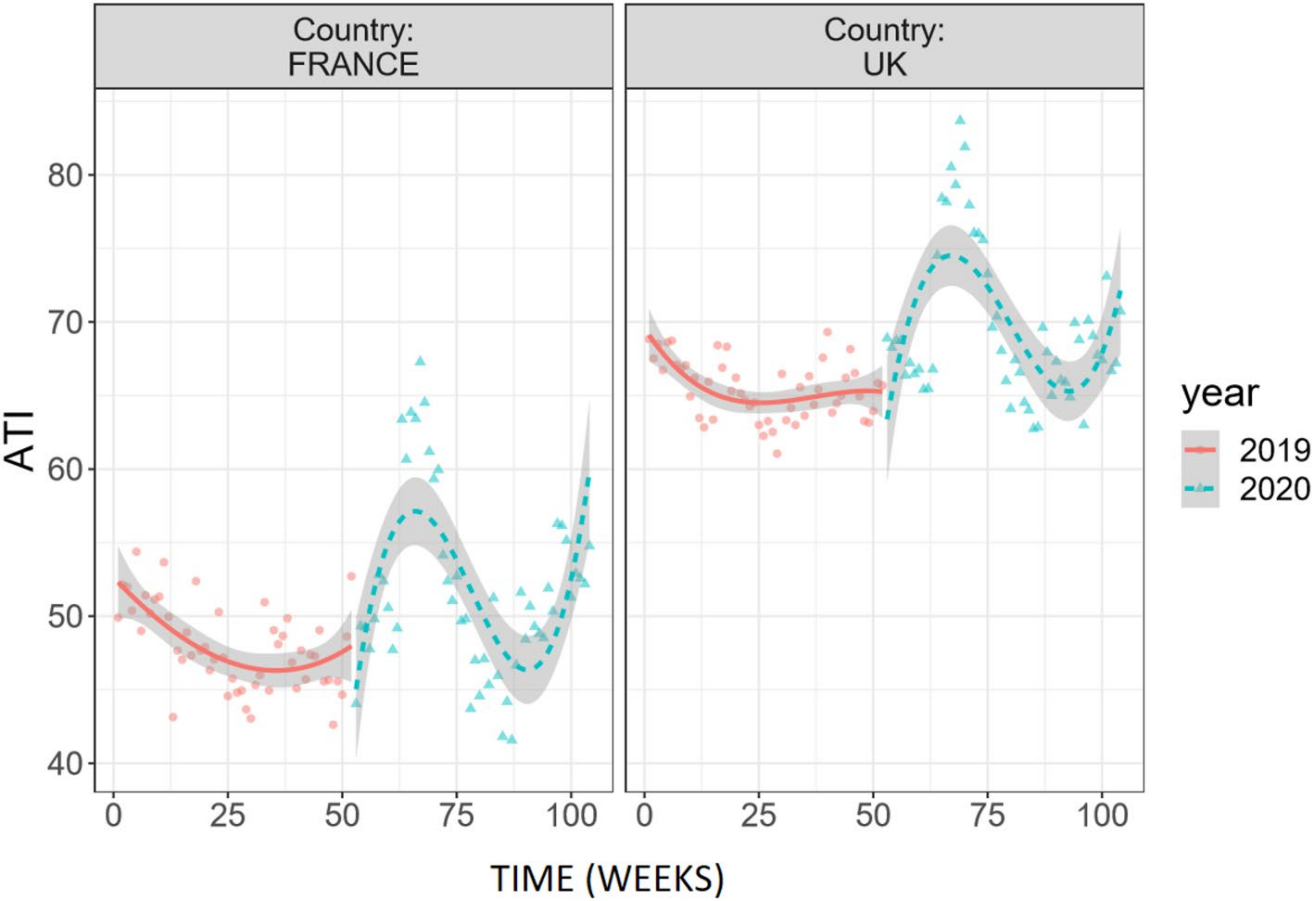
Chronogram for ATI, weekly measures across France and the UK and over the weeks from January 1st of 2019 (1) to December 30th of 2020 (n = 104). Fitted lines represent polynomial for ATI and grey areas represent SEs.

In this third analysis, we combined weekly ATI data from 2019 and 2020 (n = 104) in both France and the UK (total n = 208) As can be seen in Fig. 3, ATI dynamics are quite similar across countries, and most likely reflect a strong impact of the COVID-19 pandemic, at least during the first wave.

We then set out to test this hypothesis using Bayesian structural time-series models^38^. In short, these models allow to train Markov chain Monte Carlo algorithm to quasi-experimentally generate the estimate of an exogeneous event’s impact on a time series of interest relative to a time-series hypothesized to be unaffected by the said event. To do so, we extracted and averaged a series of 19 randomly generated words from Google Trends (using https://randomwordgenerator.com/) matched in English and French so as not to contain words with accents (e.g. “position,” “money,” “seed”, see OSF page for the full list).

**Figure 4:**
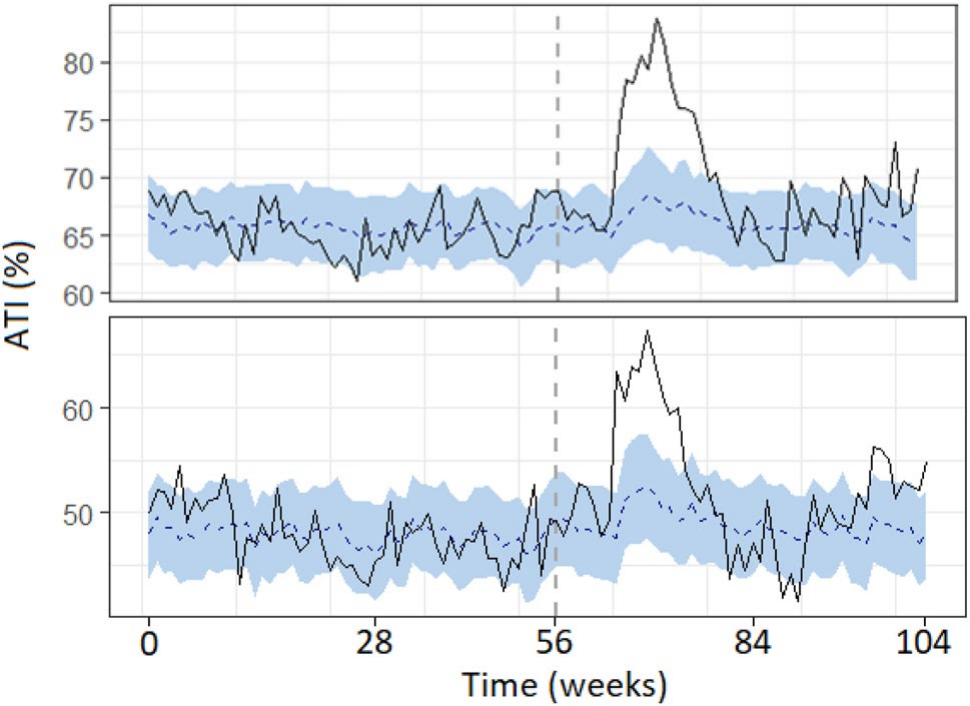
Chronogram of ATI weekly measures (dark line) relative to the synthetic predicted control time series (blue line). Measures are taken across France (below) and the UK (above) over the weeks from January 1st of 2019 (1) to December 30th of 2020 (n = 104). Vertical dashed grey lines represent the beginning of the COVID-19 pandemic.

After specifying the pre and pandemic periods based on reports of the first cases to appear in each country, (week 56 in France and 57 in the UK), we ran the Bayesian models (syntax, full tables and data available on the project OSF page). Our analyses indicate that, in line with the visual effect on Fig. 3, the pandemic did increase ATI by an absolute average 3.2%, 95%CI[2.1,4.2], *p* = 0.001 in France and by an average 3.7%, 95%CI[2.9,4.4], *p* = 0.001 in the UK (see Fig. 4).

Overall, we had strong evidence that the ATI was impacted by the pandemic, but we wished to further investigate this phenomenon. We decided to focus an analysis on data during the pandemic exclusively, factoring in both new weekly cases and deaths per million inhabitants from the COVID-19 Data Repository by the Center for Systems Science and Engineering (CSSE) at Johns Hopkins University (available at https://github.com/CSSEGISandData/COVID-19) in France and the UK (n = 96). A final mixed-effects model was therefore computed according to the following (full model available on the OSF project page):

(6) *ATI* = *1* + *cases* + *deaths* + *weeks (1 | country)*

**Figure 5:**
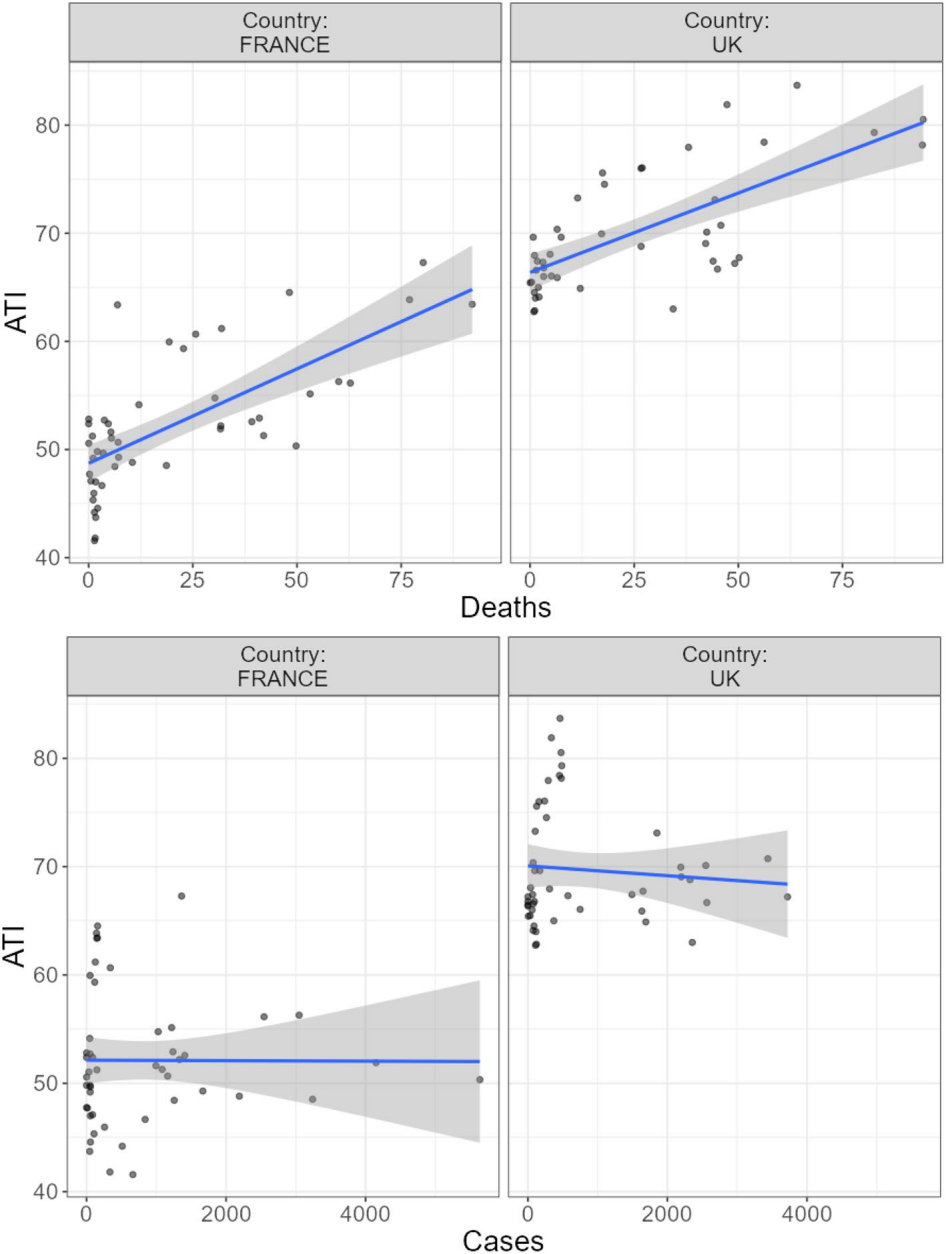
Scatterplot of ATI weekly measures as a function of COVID-19 deaths (above) and cases (below) in France (left) and the UK (right). Fitted blue lines represent linear trends for ATI and grey areas represent SEs.

Despite a general tendency to go back to baseline over the weeks, *β* =  − 0.18, 95%CI[− 0.25, − 0.10], *p* < 0.001, ATI increased significantly as a function of COVID-19 deaths *β* = 0.14, 95%CI[0.14,0.21], *p* < 0.001 , but not cases *β* =  − 0.001, 95%CI[− 0.001,0.001], *p* = 0.40 (see Fig. 5).

### Analysis 4: Cross-cultural validity: pandemic impact analysis in Japan

**Figure 6:**
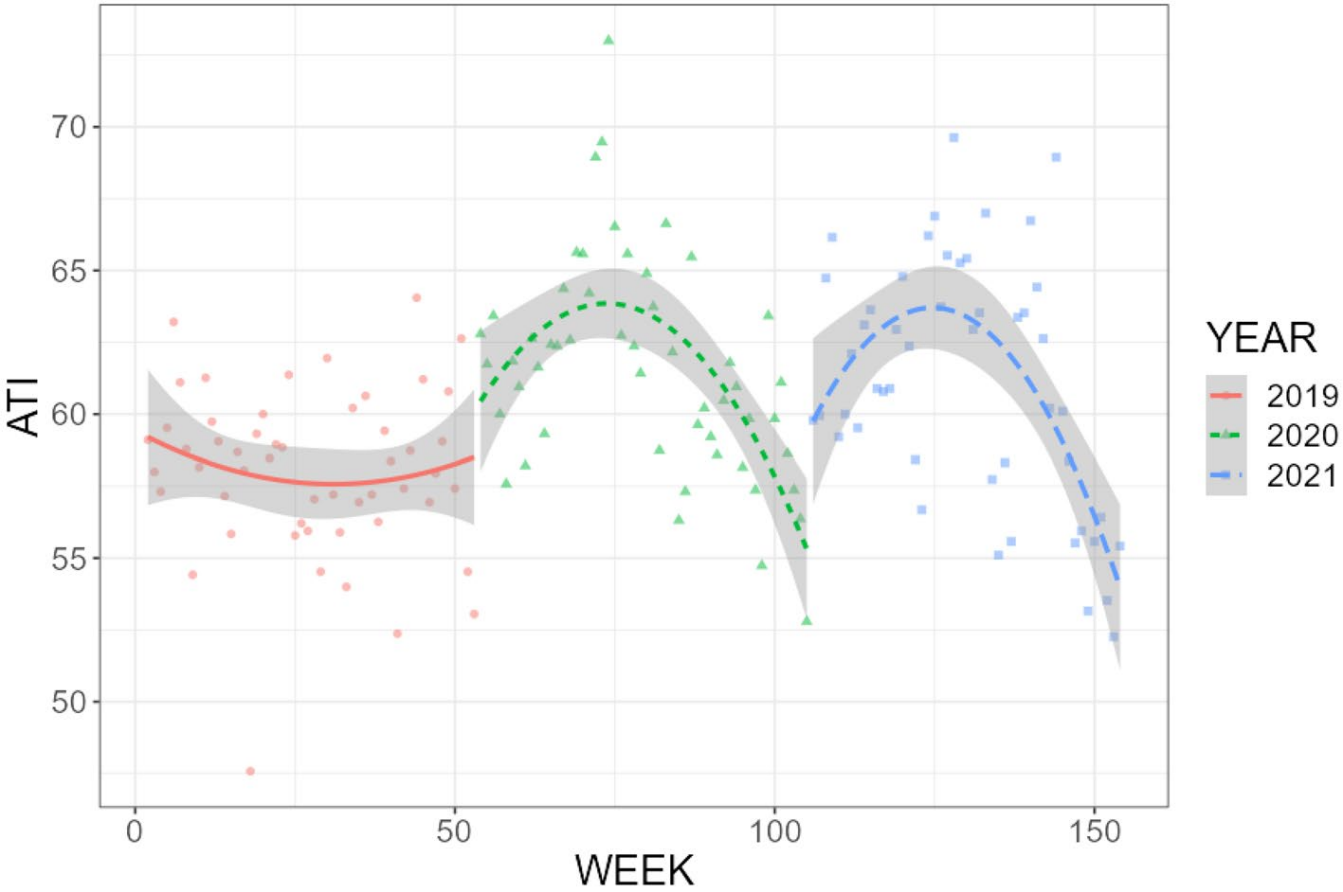
Chronogram for ATI, weekly measures across Japan over the weeks from January 1st of 2019 (1) to December 5th of 2021 (n = 153). Fitted lines represent polynomial for ATI and grey areas represent SEs.

For this last analysis, we decided to replicate our previous tests using data from Japan. We translated the ATI and the same previous randomly generated words in Japanese (see OSF page for the full list) using two validated online translators (https://translate.google.co.uk/; https://www.deepl.com/en/translator) and the help of a Japanese language expert (academic), and extracted weekly data from 2019, 2020 and 2021 (date of the manuscript’s revision for which this final this was conducted, α = 0.64, n = 153) from the Japanese cyberspace. A quick glance at the ATI dynamics for each year indicated that it was indeed sensitive to the pandemic waves (see Fig. 6).

**Figure 7:**
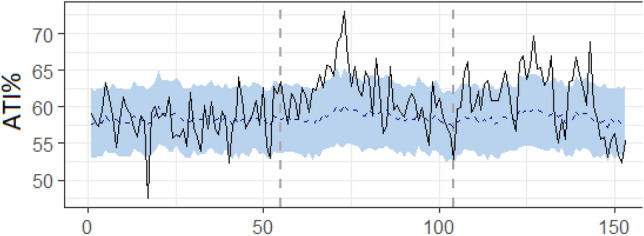
Chronogram of ATI weekly measures (dark line) relative to the synthetic predicted control time series (blue line). Measures are taken across Japan over the weeks from January 1st of 2019 (1) to December 5th of 2021 (n = 153). Vertical dashed grey lines represent the beginning of the COVID-19 pandemic.

Again, impact analyses indicated that, in line with our previous findings, the pandemic did increase ATI by an absolute average 4.9%, 95%CI[3.1,6.7], *p* = 0.001 in Japan (see Fig. 7). This, despite the different pandemic dynamics and linguistic context.

Following a similar analytic strategy as before, we decided to focus our last analysis on both new weekly cases and deaths per million inhabitants from the COVID-19 Data Repository by the Center for Systems Science and Engineering to see how these related to ATI in Japan using the following mixed-model specification (full model available on the OSF project page):

(7) *ATI* = *1* + *cases* + *deaths* + *weeks (1 | Year)*

**Figure 8:**
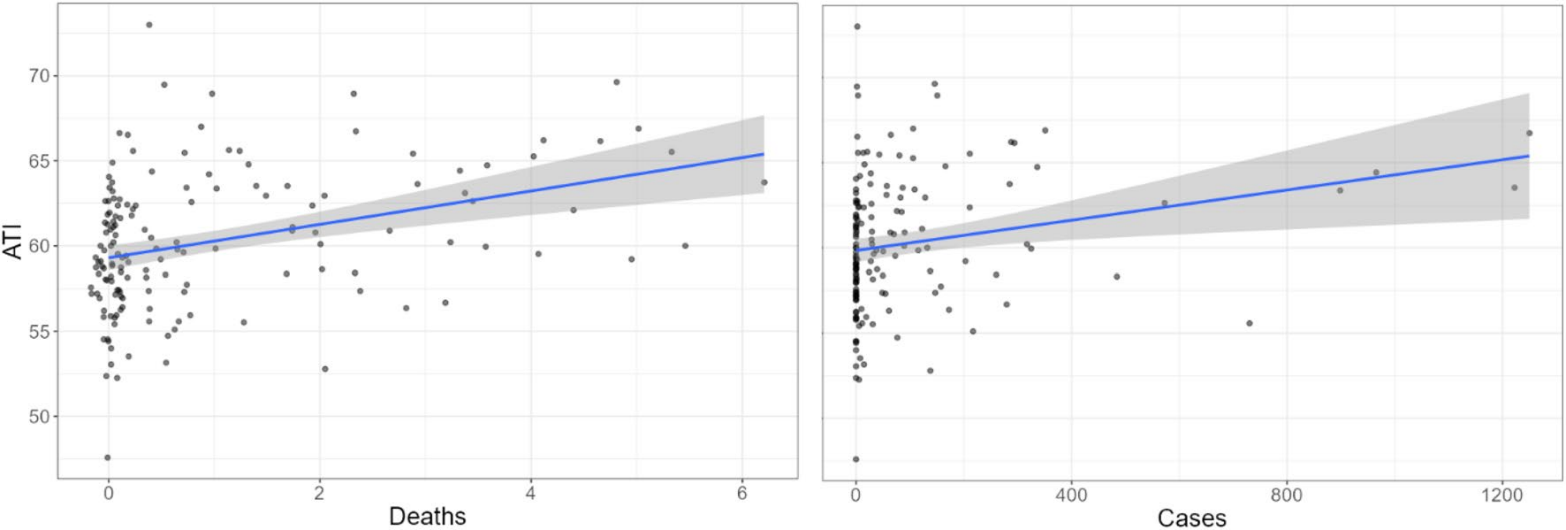
Scatterplot of ATI weekly measures as a function of COVID-19 deaths (left) and cases (right) in Japan. Fitted blue lines represent linear trends for ATI and grey areas represent SEs.

Despite a general tendency to go back to baseline over the weeks, *β* =  − 0.06, 95%CI[− 0.10, − 0.03], *p* < 0.001, ATI increased significantly as a function of COVID-19 deaths *β* = 0.90, 95%CI[0.36,0.1.44], *p* < 0.001, and this time marginally as a function of cases *β* = 0.001, 95%CI[− 0.001,0.01], *p* = 0.051 (see Fig. 8).

## Discussion

This study aimed to test the validity of a new behavioral measure of mental health based on psycholinguistic and clinical psychology theory. Across analyses, we verified that ATI was robustly associated with depression measures, as theoretically expected, with different survey operationalization, in two languages and cultural settings. Critically, ATI displayed a decrease after the first lockdown in the UK, similar to survey measures of depression (Analysis 1). In addition, ATI had a third-order polynomic behavior over time in France, just as depression measures among the population but unlike anxiety (Analysis 2). This provided further evidence for discriminant validity of the ATI.

Our impact analyses revealed that the ATI reacts just as population mental health issues was found to react during the pandemic, with a steep increase in the first wave and a decline as individuals adjust, to degrade once more with the second incoming wave (Analysis 3). The differences in ATI in 2020 compared to 2019 are also in line with estimates from various survey studies in the US and the UK^2,9,10^. These patterns were observed in Japan too, providing cross-cultural validity to ATI as a measure of population-level depression.

In addition, the main driver of ATI increases seems to be COVID-19 deaths in the population (although a marginal influence of cases was also noted in Japan), in line with what could be expected in terms of population-level depression increases as individuals lose close relatives. On the other hand, more substantial work needs to be conducted to disentangle conjoint economic (e.g. unemployment), policy (e.g. lockdown) and psychosocial (e.g. emotion contagion) effects of the pandemic on population mental health.

Overall, we believe that the ATI could be used as a potent multipurpose cost-effective monitor of population mental health. As discussed in earlier studies^25^, such a tool could be used to assess not only pandemic mental health consequences, but also the effect of public mental health policies, like suicide prevention campaigns. Retrospective analysis on local policies or targeted geographical areas may help discover community resilience factors of interest. Unusual ATI behavior in areas of interest may help diagnose at-risk communities and assist allocation of economic resources dedicated to improve population mental health outcomes. Sudden changes in ATI could also help discover emergent pandemics (e.g. substance abuse) before the effects are seen on hospital admissions for overdose. Finally, discrepancies between ATI and survey data may also be used to highlight methodological issues and misestimates of population mental health through traditional survey means.

### Limitations

Nonetheless, the results of the present study remain constrained by four main limitations. First and foremost, it should be noted that search volume data is measured for all users who use the certain search terms—whom we do not have information about. The sample of users providing remains relatively unknown in terms of demographics, socio-economic, ethnic psychological, genetic, and pathological background.

Relatedly, the longitudinal survey study used to test the validity of the ATI in the UK presents non-negligible deviations from the country’s population characteristics. This bias can even be magnified due to the study’s very large sample size^39^. Although this bias is partly compensated by weighting and a replication of the ATI’s validity among representative samples in France, we cannot not definitely rule out its potential influence on our results.

The uncertainty regarding the characteristics of Google Trends users and the sometimes-non representative survey samples also means that there may be a discrepancy between the populations underlying both types of data. This discrepancy is not a threat for the study’s internal validity (i.e. the relationships between both indicators) but could bias point estimates of mental health issues in the population from both instruments.

Likewise, the different instruments used to tap into anxiety/depression in both survey studies pose limits to what can be inferred when comparing the populations from both countries. It must be noted that different cutoff scores (e.g. at high and not moderate levels of depression) might have yielded different results with the ATI. Still, converging evidence obtained for the validity of the ATI across this variety of measures and samples is an indication of its ecological validity^40^.

Finally, Japanese translation cannot be matched one-to-one with English/French. For instance, adverbs may only be interpreted as such depending on their position in a sentence. Also, Japanese use two different character systems. Our translated version—due to constraints regarding meaning—makes use of words from both, but some may be used less frequently than others. These elements may explain the lower reliability of the Japanese ATI, although converging results across linguistic contexts provide first evidence for ATI’s cross-cultural validity.

## Conclusion

Although more work needs to be conducted to guarantee the external validity of ATI as a measure of population mental health in other settings and languages—and within the boundaries of the above-mentioned limitations, we believe to have established strong evidence in favor of considering the ATI for research and applied purposes in public mental health in Western European countries.
